# Acute vertigo and dizziness: the diagnostic potential of static posturography in the neurological emergency department

**DOI:** 10.1007/s00415-026-13823-z

**Published:** 2026-04-21

**Authors:** Laurin Schappe, HeimigEmily Heimig, Daniel Janitschke, Maximilian Uwe Friedrich, Patrik Theodor Nerdal, Nicolas Rohmann, Frauke Röll, Daniel Martens, Mathias Fousse, Jakob Stögbauer, Gudrun Wagenpfeil, Yaroslav Winter, Sergiu Groppa, Ulrich Dillmann

**Affiliations:** 1 Department of Neurology, Saarland University Medical Center, Kirrberger Straße, Building 90, 66421 Homburg, Saarland Germany; 2 Department of Neurology, University Hospital Ulm, 89081 Ulm, Germany; 3 Department of Neurology, University Hospital Schleswig-Holstein and Kiel University, 24105 Kiel, Germany; 4 Institute of Medical Biometry, Epidemiology and Medical Informatics (IMBEI), Saarland University Medical Center, 66421 Homburg, Saarland Germany

**Keywords:** Emergency room, Vertigo, Dizziness, Posturography, Functional dizziness

## Abstract

**Background:**

Vertigo and dizziness are common in the emergency room, caused by a broad spectrum of conditions ranging from life-threatening to non-organic causes such as functional dizziness. The latter is often underdiagnosed, leading to chronic symptoms and high costs.

**Objective:**

To evaluate the diagnostic value of a short dual-task static posturography (sPG) protocol in patients with acute vertigo and dizziness in the neurological emergency department.

**Methods:**

We prospectively assessed 211 patients using sPG. Established patterns (normal, functional, cerebellar, sensory ataxic) were rated on a 5-point scale by two blinded experts. Interrater agreement and non-parametric analyses were performed, and diagnostic classifications were compared with and without sPG.

**Results:**

Interrater agreement was high (Kappa = 0.965). sPG identified functional patterns in 29.3% of previously unclear vertigo/dizziness cases, leading to a highly significant increase in the diagnosis of functional dizziness (*p* < 0.001). Functional patterns were also frequently observed in organic diagnoses, particularly in episodic vestibular disorders. Furthermore, by detecting cerebellar patterns, sPG identified 20% more cases of central vestibular disorders (from 45 to 54 cases).

**Conclusion:**

sPG offers a rapid, cost-effective tool to identify functional dizziness in the emergency setting, complementing standard clinical assessment. It facilitates early recognition of functional dizziness and may reduce underdiagnosis and prolonged symptom chronification.

**Supplementary Information:**

The online version contains supplementary material available at 10.1007/s00415-026-13823-z.

## Background

Vertigo and dizziness are among the most common presenting symptoms in the emergency room [1–3]. Possible causes range from potentially life-threatening diseases such as stroke to more benign inner ear conditions such as peripheral paroxysmal positional vertigo (PPPV), or complex neuropsychiatric conditions such as functional dizziness [1–3]. In specialized outpatient clinics, functional dizziness constitutes the most common cause for presentation [4], while it is the fifth most common cause for presentation in the general emergency room [2, 3]. However, functional dizziness remains underdiagnosed outside of specialized clinics, often leading to chronification and a high risk of excessive diagnostic measures, which cause a significant economic burden [5–7]. Diagnostic tools such as video-head impulse test and posturography, supplementing the clinical examination of vestibulo-ocular reflex and postural control, have been shown to provide crucial diagnostic clues [8, 9]. Static posturography (sPG) is a method which, albeit receiving less attention in vertigo and dizziness management, has shown diagnostic utility in balance disorders, such as cerebellar or sensory ataxias [10, 11]. Furthermore, paired with dual-tasking procedures, sPG can provide critical clues towards a so-called functional cause of chronic imbalance, defining functional dizziness [12, 13]. One such functional finding in functional dizziness is increased sway during simple tasks, with paradoxical improvement during more complex dual-task conditions, such as mental arithmetic [14]. While these data underscore the diagnostic value of sPG in chronic cases of imbalance, its utility in detecting functional findings in acute cases of vertigo and dizziness is unclear. Hence, the aim of this study is to test the diagnostic value of a short dual-task sPG-based protocol in patients presenting with acute vertigo and dizziness in the emergency room.

## Methods

### Participants and protocol

Over the course of 1 year, 222 patients presented with a chief complaint of vertigo and dizziness to our neurological emergency department at Saarland University Medical Center. Exclusion criteria for the study were inability to stand unaided for 3 minutes or excessive body weight. Therefore, 11 cases were excluded and 211 consecutive patients were included in the study. In our cross-sectional study, each patient’s medical history was obtained and a standardized clinical examination was performed. Our examination included the National Institutes of Health Stroke Scale (NIHSS) and the video-oculography-assisted HINTS plus examination, which consists of the head impulse test, assessment of nystagmus with Frenzel goggles, evaluation of skew deviation, and hearing test by finger rub [15]. The ABCD2 score (range 0–7), incorporating age, blood pressure, clinical features, symptom duration, and diabetes mellitus, was used to assess early stroke risk [16]. In addition, positioning maneuvers such as the diagnostic Sémont maneuver and supine roll test, the head-shaking test, and the Romberg test were conducted. All patients underwent blood pressure measurement, electrocardiography, blood sampling and cranial computed tomography. By central HINTS findings or an ABCD2 score ≥ 3 [17], cranial computed tomography was performed either with contrast enhancement or supplemented by duplex ultrasonography. Patients with suspected stroke were admitted for inpatient evaluation and, when symptoms persisted for more than 24 h, underwent additional cranial magnetic resonance imaging. In cases of suspected inflammatory etiology, lumbar puncture was performed.

Diagnoses were made in accordance with the current national guideline ‘Vestibular Dysfunctions’ [14], the International Classification of Vestibular Disorders of the Bárány Society, as well as the ICD-11. Additionally, patients were diagnosed with sensory neuropathy if they presented with symptomatic sensory ataxia as their main symptom along with additional clinical signs of polyneuropathy. We summarized hemodynamic orthostatic dizziness/vertigo, hypertensive derailment, cardiac arrhythmia, electrolyte disturbances and intoxication into a singular category named “other”. Cases where symptoms could not clearly be attributed to one of these causes were labeled as “vertigo/dizziness syndromes of unclear origin”.

**Fig. 1:**
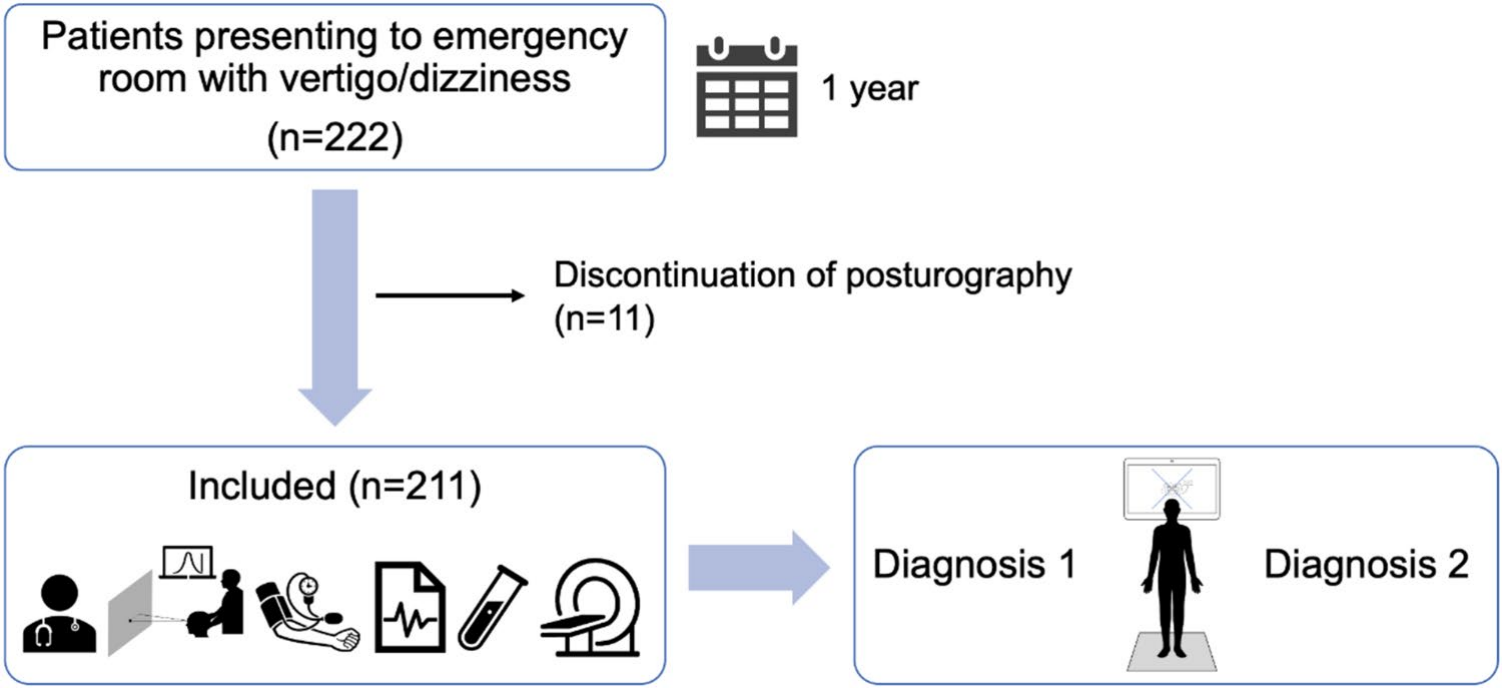
Study protocol. Patients were recruited from March 27, 2019, to March 9, 2020. Figure prepared using Keynote and PowerPoint, with additional support from ChatGPT (OpenAI, version January 2026)

Based on the examinations described above and current clinical guidelines, a diagnosis was assigned for each patient. An sPG was then performed and independently evaluated in a double-blinded manner by two electrophysiology specialists (UD and YW) to determine whether the findings were consistent with the diagnosis made in the emergency department or indicated an alternative diagnosis (Fig. 1).

### Static posturography

Data were recorded using a stabilometer platform (Jaeger-Toennies, Hochberg, Germany) at a sampling rate of 1000 Hz, with a measurement duration of 60 s per trial. Postural stability was assessed using the center of pressure (CoP) and its sway area, which represents the surface covered by the CoP trajectory during the measurement period. Patients stood freely with arms hanging naturally by their sides, positioned two meters away from a wall, with a video monitor attached at eye level. Measurements were performed under eyes-open, eyes-closed and visual feedback conditions in randomized order. Visual feedback was assessed using a dual-task approach: the CoP, which was visualized on the opposite video monitor, was to be held in the center of a square.

The sway area was calculated using the 95% confidence ellipse. Standard deviations of CoP trajectories in the anteroposterior and mediolateral planes were computed for each task. To account for inter-individual differences in body mass, forces recorded by the platform were normalized to body weight and expressed as a percentage of body weight (%BW): F_%BW_ = F_measured_/F_bodyweight_ × 100. In addition, frequency-domain characteristics of CoP fluctuations were analyzed using Fourier analysis. In-house, age-matched normative data was used for interpretation. Results were visualized using scatter plots.

**Fig. 2:**
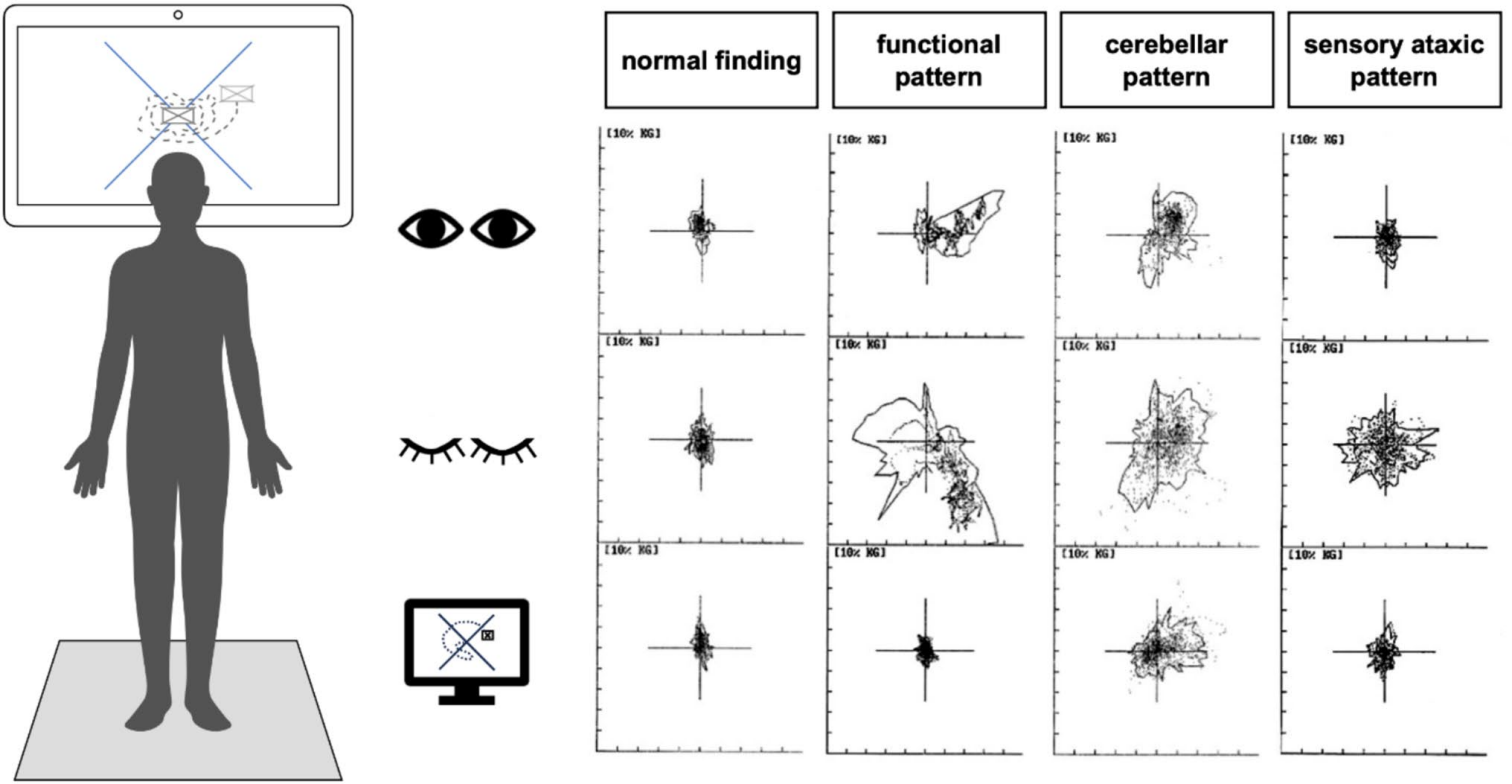
Static posturography and typical findings. Every millisecond, the center of pressure (CoP) is continuously recorded on the posturographic force plate in both, the anteroposterior and mediolateral directions. These measurements are then compiled into a position histogram, illustrating how often the CoP occupies specific areas and providing a visualization of balance stability and sway patterns. Figure prepared using Keynote and PowerPoint

Standardized sPG patterns, previously established and quantified for cerebellar and sensory ataxias, were utilized in this study [8, 10, 11, 18]. Furthermore, patients with functional dizziness are known to exhibit normal to improved stability under dual-task conditions [12, 13]. Figure 2 shows examples for typical sPG findings.

Posturography data were independently evaluated in a double-blinded fashion by two electrophysiology experts (UD and YW). Each patient’s posturography patterns—normal, functional, cerebellar, and sensory ataxic—were assessed using an ordinal 5-point scale. The scale was defined as follows: 1 = no evidence of the pattern, 2 = questionable indication of the pattern, 3 = pattern is evident but not clearly expressed, 4 = pattern is clearly expressed, and 5 = pattern is strongly expressed.

### Statistical analysis

Interrater agreement was quantified using quadratically weighted Cohen’s Kappa. Group comparisons of sPG measurements across predefined pattern groups were performed using the Kruskal-Wallis test (one-way ANOVA on ranks). Post hoc pairwise comparisons were adjusted for multiple testing using the Bonferroni correction. To evaluate the diagnostic impact of sPG, a pre-post classification approach was applied to assess changes in diagnostic distributions following sPG assessment. Changes in diagnostic assignment were evaluated per patient, and pre-post diagnostic classifications were compared using the exact McNemar test for paired categorical data. All data were inspected for outliers, defined as values exceeding ± 3 standard deviations from the group mean. Six measurements were excluded and three patients were unable to complete the sPG feedback task because they did not understand the instructions; their other sPG measurements were still included in the analysis. Non-parametric analyses were used to minimize the influence of extreme values. Normality of continuous variables was assessed using the Shapiro–Wilk test and visual inspection of Q-Q plots. Age and sex were explored in stratified descriptive analyses to assess potential confounding effects. Statistical analyses were performed using R (version 2025.09.2 + 418; R Core Team) and SPSS (version 29.0.0.0). A significance level of p < 0.05 was applied throughout.

## Results

### Demographic data

83 male and 128 female patients were included in this study, resulting in a total of 211 patients (18–90 years, mean = 53.7, standard deviation (SD) = 17.8 years). sPG findings included functional (24–84 years, mean = 46, SD = 18 years), cerebellar (27–83 years, mean = 65, SD = 15 years), sensory patterns (18–90 years, mean = 58, SD = 18 years) und normal findings (24–84 years, mean = 52, SD = 15 years).

### Posturographic data

The ratings demonstrated very high agreement between the two experts (Kappa = 0.965, *p* < 0.001), confirming the statistical reliability of the sPG assessments. Taking into account only clearly and strongly expressed patterns (4 and 5 on the ordinal 5-point scale), 81 patients had normal findings, 44 patients showed a functional pattern, 22 patients had a cerebellar pattern, and 20 patients had a sensory ataxic pattern. In 44 patients, the sPG findings showed only minor deviations from normative data across all tasks, precluding clear assignment to a specific pattern.

**Fig. 3:**
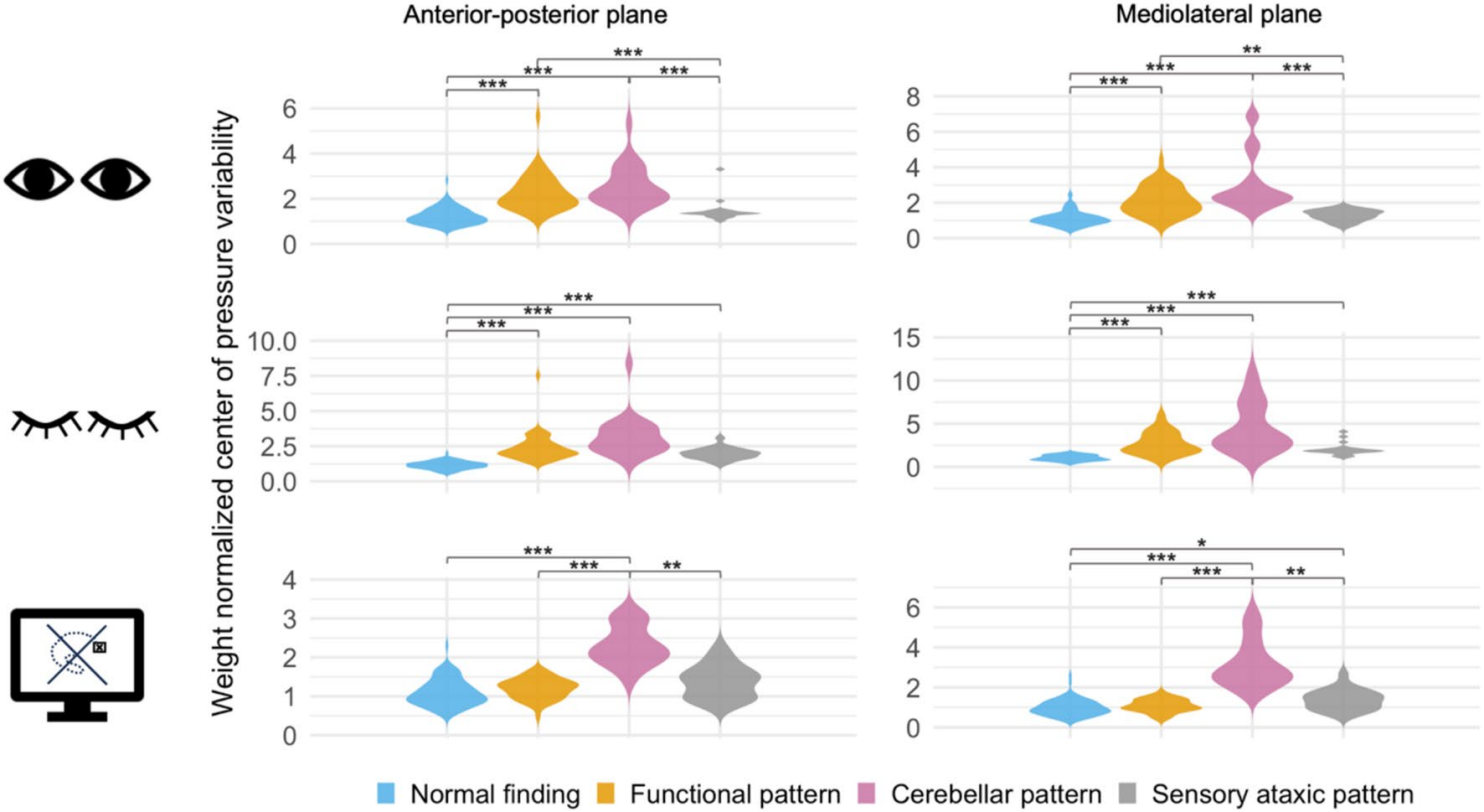
Violin plots showing the weight normalized center of pressure (CoP) variability in the anteroposterior and mediolateral planes for different posturographic patterns. Therefore, the standard deviations of CoP trajectories in the anteroposterior and mediolateral planes were calculated for each patient and task. Note the different y-axis scales, which reflect the varying normative values across the conditions. Except for 44 patients with no clear assignment to any specific pattern, all relevant sPG patterns are shown in the figure: normal (*n* = 81), functional (*n* = 44), cerebellar (*n* = 22), and sensory ataxic (*n* = 20). Significance threshold: *p < 0.05, **p < 0.01, ***p < 0.001. Figure generated using R

Figure 3 shows the normalized mean CoP variability in the anteroposterior and mediolateral planes for different sPG patterns. The plots illustrate the distribution of CoP measures.

Patients with functional and cerebellar patterns both exhibited increased CoP variability in eyes-open and eyes-closed tasks. Violin plots illustrate that, despite these pathological values, the distributions largely overlapped. The only statistically significant difference between the two patterns was observed in the visual feedback condition ( *p* < 0.001), highlighting that visual feedback was the key feature distinguishing functional from cerebellar patterns: whereas in cerebellar patterns the values lay outside the normal range across all conditions, visual feedback remained within the normal range in patients with functional patterns. In sensory ataxic patients, CoP variability was increased with eyes closed, predominantly in the mediolateral plane. A significant difference from normal was also observed in the lateral feedback condition (*p* = 0.011).

### Diagnosis distribution with and without static posturography assessment

The most common diagnoses were central vestibular vertigo/dizziness (CVV), PPPV, acute unilateral vestibulopathy (AUVP), and vestibular migraine (VM) (Table 1). 6.2% of patients (13) had orthostatic or other internal medicine causes, and 3.8% (8) met criteria for functional dizziness.

**Table 1:** Diagnoses with and without implementation of static posturography

Diagnoses	Without posturography (*n*)	With posturography (*n*)	Exact two-sided McNemar test (*p* values)
Functional dizziness	8	25	< 0.001
Central vestibular vertigo/dizziness	45	51	0.0313
Vertigo/dizziness syndromes of unclear origin	58^★^	37	< 0.001
Peripheral paroxysmal positional vertigo	35^✦^	34	1.0000
Acute unilateral vestibulopathy	20	20	Test not applicable
Vestibular migraine	18	18	Test not applicable
Menière’s disease	7^✤^	6	1.0000
Bilateral vestibulopathy	5^❖^	3	0.5000
Sensory neuropathy	2	4	0.5000
Other	13	13	Test not applicable
Total	211	211	

^★^ = 17 functional pattern, 2 cerebellar pattern, 2 sensory ataxic pattern, ^✦^ = 1 cerebellar pattern, ^✤^ = 1 cerebellar pattern, ^❖^ = 2 cerebellar pattern

The implementation of sPG led to a highly significant increase in the diagnosis of functional dizziness (*p* < 0.001), making it the third most common diagnosis in the cohort. Functional patterns were identified in 29.3% (*n* = 17) of previously unclear cases of vertigo or dizziness, all of whom exhibited no abnormalities in any other assessments. This allowed functional dizziness to be diagnosed significantly more frequently.

Furthermore, by detecting cerebellar patterns, sPG identified a 20% increase in cases of CVV (from 45 to 54 cases), which was statistically significant (*p* = 0.0313). Cerebellar ataxia was observed in two patients with vertigo/dizziness syndromes of unclear origin and in several diagnostic groups, including one patient initially diagnosed with PPPV, one initially diagnosed with Menière’s disease (MD) and two initially diagnosed with bilateral vestibulopathy (BVP).

Overall, 27.5% (58) of cases remained initially unclassified. Implementation of sPG clarified 36.2% (*n* = 21) of these cases, resulting in a significant impact (p > 0.001): 29.3% (17) were assigned to a functional pattern, 3.4% (2) were assigned to a cerebellar pattern, and 3.4% (2) were assigned to a sensory ataxic pattern.

### Occurrence of functional patterns in main organic diagnoses

**Fig. 4:**
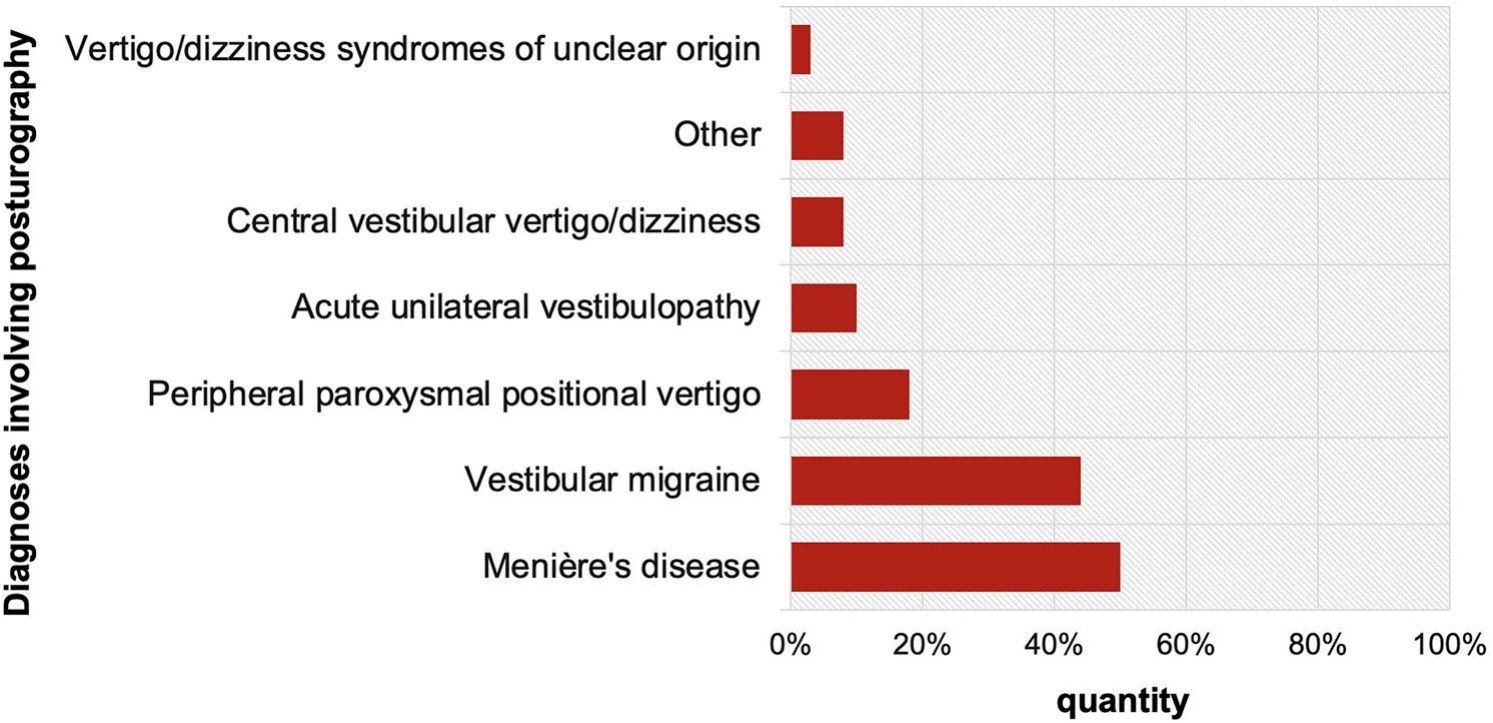
*Proportion of a functional pattern across different diagnostic groups,* excluding those with functional dizziness, following sPG implementation as shown in Table 1. Figure created using Excel

Analysis of all sway patterns further showed that functional patterns may also be present in patients with a main organic diagnosis

 (Figure 4). A functional pattern was observed in 50% (3 of 6) of patients with MD, 44.4% (8 of 18) with VM, 17.6% (6 of 34) with PPPV, 10% (2 of 20) with AUVP, and 7.8% (4 of 51) with CVV. Notably, episodic vestibular disorders showed the highest prevalence of functional patterns.

## Discussion

CoP analysis in dual-task posturography enables early identification of a functional pattern in acute vertigo and dizziness. This is particularly important, as our data show that early detection of a functional pattern was present in 29.3% (17 of 58) of patients with vertigo/dizziness syndromes of unclear origin (Table 1). These patients had previously undergone comprehensive testing, including clinical examination, video-based head impulse testing, cranial imaging, and laboratory work, without revealing any abnormalities. Only sPG provided a possible indication of the underlying cause of vertigo and dizziness. In standard practice, such patients typically leave the emergency department without a definitive diagnosis and undergo further outpatient evaluation. In doing so, about half of the patients see primary care physicians and the other half consult specialists; only 70% of patients with vestibular vertigo receive an explicit diagnosis [7]. According to diagnostic criteria, a diagnosis of functional dizziness can generally be made only after a three-month period of persisting symptoms [19]. Delaying functional dizziness diagnosis is met risks symptom chronification as well as the risk of excessive diagnostic measures [5]. Early implementation of sPG could therefore identify patients at risk for functional dizziness, potentially offering a substantial clinical advantage.

An advantage that may be particularly important is that, in our study, functional dizziness was clearly underdiagnosed without sPG. The use of sPG for detecting functional dizziness produced a statistically significant increase (Table 1): while only 3.8% of cases (8 of 211) were identified as functional dizziness without sPG, this proportion rose to 11.8% (25 of 211) when functional sPG patterns were considered. Thus, functional dizziness emerged as a very common diagnosis and may be more prevalent in the emergency department than previously reported [2, 3]. Time in the emergency department is often limited for a thorough history, and commonly used clinical scores such as the NIHSS, ABCD2, or HINTS do not include assessments capable of differentiating functional disorders, such as dual-task testing. In this context, dual-task sPG with simple CoP pattern analysis provides a rapid and cost-effective method to detect and record clinically relevant functional findings.

Additionally, our study demonstrates that functional involvement, typically identified through comprehensive clinical evaluation, supported in studies by psychometric assessment [20], can be detected efficiently using sPG within just a few minutes. Functional dizziness may occur both as a primary diagnosis and as a secondary psychosomatic comorbidity in patients with a main organic diagnosis [19, 21]. Psychiatric comorbidities, particularly anxiety and depression, have been suggested to contribute to the range of symptoms, especially in Meniere’s disease and vestibular migraine [20], and anxiety-related alterations in postural strategies likely play a role pathophysiologically [5, 8]. In our cohort, functional patterns were observed in patients with primary organic diagnoses, including MD, VM, PPPV, AUVP, and CVV (Fig. 4). Another study also showed that posturography is the most sensitive diagnostic tool for detecting secondary functional dizziness, with PPPV and VM being the most common somatic triggers [22]. In our study, MD may be overrepresented, possibly due to the small sample size. Notably, episodic vestibular disorders also exhibited the highest prevalence of functional patterns, suggesting that secondary functional involvement tends to emerge subacutely. This underlines the importance of determining the optimal timing for an early interdisciplinary therapeutic approach that also includes psychosomatic treatment components.

Regarding cerebellar and sensory ataxic patterns, sPG contributed to clarifying previously unclear diagnoses in two cases for each pattern (Table 1). Ultimately, sPG did not provide a significant additional benefit for identifying sensory ataxia, whereas significant diagnostic changes were observed for the detection of cerebellar ataxia (Table 1). These findings, however, should be interpreted with caution. They are based on only six cases in which the diagnosis was revised to CVV following sPG assessment. Among these, two patients had previously been diagnosed with BVP. In the context of CANVAS syndrome, cerebellar ataxia may indeed coexist with peripheral vestibulopathy, meaning that a change from BVP to a CVV diagnosis does not represent a misdiagnosis; rather, both conditions may be present. Recent studies have shown that clinical gait and stance tests, including posturography, do not reliably differentiate central from peripheral etiologies in isolated acute vertigo and dizziness among patients with mild to moderate symptom burden [23].

Like Hadzhikolev et al., methodologically most studies have focused primarily on differentiating between peripheral and central causes. Our study demonstrates that it is not only important to make this distinction, but also to identify functional causes. Accordingly, more research is needed with detection of functional dizziness as a primary endpoint. In particular, follow-up studies are warranted to determine whether patients exhibiting a functional pattern in sPG meet the diagnostic criteria for functional dizziness after 3 months and ultimately receive this diagnosis.

## Restrictions

Our study was conducted in a neurological emergency department; however, we consider vertigo and dizziness an interdisciplinary leading symptom and in unexplained cases the cause might lie in another specialty. Relevant neurological causes were ruled out by diagnostics in our emergency department and further outpatient clarification could have been indicated for these patients, at least on this basis.

In 44 cases, no clear assignment to a specific pattern could be made, because sPG findings showed only minor deviations from normative data across all tasks. This represents 20.9% of cases in the study for which sPG could not provide a clear classification as normal, functional, or ataxic. Conversely, in 40.8% of cases, these patterns provided a clear indication of the underlying cause of vertigo and dizziness.

## Conclusion

Dual-task posturography improves the classification of acute vertigo and dizziness syndromes in the emergency department, particularly enabling early identification of a functional pattern. Therefore, early implementation of sPG could identify patients at risk for functional dizziness, potentially offering a substantial clinical advantage. In our study sPG enabled the identification of functional patterns in patients with previously unclear diagnoses, leading to a highly significant increase in functional dizziness and highlighting patients at risk for chronic functional dizziness. Functional patterns were also observed in patients with primary organic diagnoses, particularly in episodic vestibular disorders, indicating that secondary functional involvement may emerge subacutely and emphasizing the importance of timely treatment.

## Supplementary Information

Below is the link to the electronic supplementary material.

Supplementary file1 (EPS 16278 KB)

## Data Availability

All figures have associated raw data. The data that support the findings of this study are available from the corresponding author upon reasonable request.

## Abbreviations

AUVP: Acute unilateral vestibulopathy
BVP: Bilateral vestibulopathy
CoP: Center of pressure
CVV: Central vestibular vertigo/dizziness
HINTS plus: Acronym for the following 3 oculomotor tests: head impulse test, nystagmus and test of skew and test of hearing loss
ICD-11: International Classification of Diseases 11th Revision
MD: Menière’s disease
NIHSS: National Institutes of Health Stroke Scale
PPPV: Peripheral paroxysmal positional vertigo/benign paroxysmal positional vertigo
sPG: Static posturography
SD: Standard deviation
VM: Vestibular migraine
